# Alveolar Proteinosis Secondary to *M. tuberculosis*, in a Patient with Transient CD4 Lymphocytopenia Due to *Cryptococcus neoformans* Infection: First Case in the Literature

**DOI:** 10.3390/idr14020021

**Published:** 2022-03-04

**Authors:** Daniel Augusto Martin Arsanios, Diego Alejandro Cubides-Díaz, Natalia Muñoz-Angulo, Maria Alejandra Perez-Hernandez, Marlyn Zamora Posada, Mónica Briceño Torres, Carlos Mauricio Calderón Vargas

**Affiliations:** 1 Internal Medicine Resident, Universidad de la Sabana, Chía 250001, Colombia; danielmaar@unisabana.edu.co (D.A.M.A.); diegocudi@unisabana.edu.co (D.A.C.-D.); mariaperher@unisabana.edu.co (M.A.P.-H.); marlinzapo@unisabana.edu.co (M.Z.P.); monica.briceno@unisabana.edu.co (M.B.T.); 2 General Physician, Universidad el Bosque, Bogotá 110111, Colombia; 3 Hospital Universitario la Samaritana, Bogotá 110111, Colombia; caldecal@gmail.com

**Keywords:** lymphocytopenia, cryptococcal infection, interstitial lung disease, *Mycobacterium tuberculosis* infection, immune reconstitution

## Abstract

Transient CD4 lymphocytopenia is defined as the transitory presence of CD4+ T lymphocyte fewer than 300 cells/mm^3^ or less than 20% of T cells without HIV infection. It can occur due to multiple causes; however, it is rare for it to occur due to opportunistic infections. Few cases have been described in the literature where antimicrobial treatment normalizes the CD4 count, being more frequent in *Mycobacterium tuberculosis* infections. To date, this phenomenon has not been described in *Cryptococcus neoformans* infections. This would be the first reported case according to our knowledge, of a patient who normalizes CD4 count after antifungal treatment, later developing alveolar proteinosis due to *M. Tuberculosis.*

## 1. Introduction

The way the immune system responds to infections is still under continuous investigation since there are many features not yet understood. The current conception that opportunistic infections are the consequence of an immunosuppressive condition might not always be true, and occasionally infectious stimuli lead to transient immunosuppressive states, such as CD4 lymphocytopenia [1,2]. Transient CD4 lymphocytopenia was defined as the transitory presence of CD4+ T lymphocyte fewer than 300 cells/mm^3^ or less than 20% of T cells without HIV, this extrapolated from the 1992 CDC definition, without having a stipulated recovery time [1]. Transient CD4 lymphocytopenia associated with infectious conditions has been described previously, but so far not reported with *Cryptococcus neoformans* infections [2].

Alveolar proteinosis is a lung disease characterized by the accumulation of lipoproteinaceous material and dysregulation of macrophages in the alveoli. Any trigger that alters macrophage function can cause this condition, including genetic, autoimmune, toxic, infectious, and neoplastic causes. Macrophage dysfunction may also lead to a state of immunosuppression and a special susceptibility to pathogens whose elimination requires a strong innate immune response, such as *M. tuberculosis* [3]. Differentiating between the cause and the consequences of alveolar proteinosis could be challenging in the clinical scenario, and response to antimicrobial treatment could help to distinguish the initial trigger. The present would be among the first 15 cases to our knowledge describing the association between *M. tuberculosis* and alveolar proteinosis [4,5,6,7,8,9,10,11,12,13,14,15].

## 2. Case Report

A 32-year-old man, farmer with exposure to pigeon’s droppings and barn hay, was admitted in November-2020 for progressive dyspnea and dry cough of 2 months of evolution, exacerbated a week before admission. He did not present fever, myalgias, anosmia, dysgeusia, headache, or another symptomatology. He had a medical history of meningeal cryptococcosis and CD4 lymphocytopenia (264 cells/mm^3^) diagnosed in 2019, managed with induction scheme (amphotericin b + flucytosine) and currently on maintenance scheme with fluconazole.

**Figure 1:**
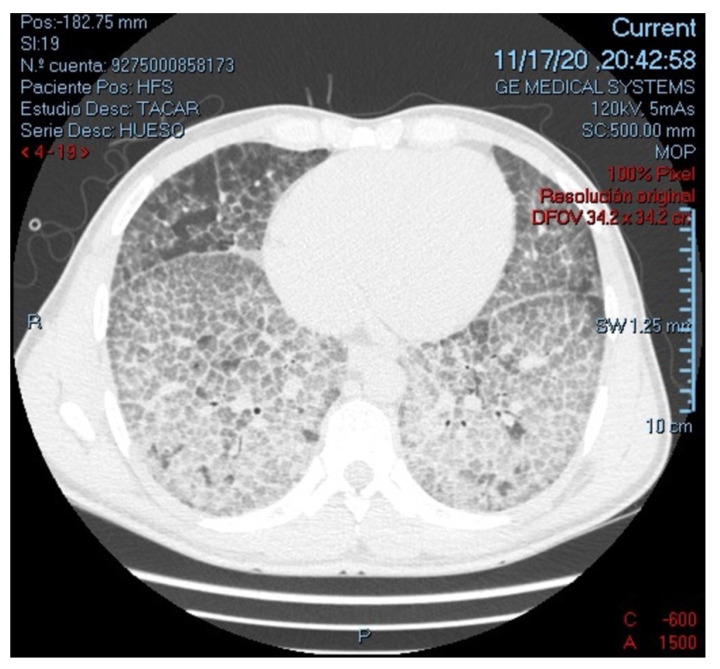
High-resolution computed tomography of the chest (parenchymal view), showing multiple ground-glass opacities with thickening of interlobular septae, configuring an extensive characteristic “Crazy-paving” pattern in both lungs.

On physical examination he was, tachycardic, tachypneic, febrile, hypoxemic, with decreased intensity of lung sounds on both sides and requiring use of accessory breathing muscles. As the only additional finding, primary gaze was deconjugated with bilateral exophoria and limitation for adduction of both eyes, findings related to the sequelae of previous meningeal cryptococcosis. Laboratories on admission presented normal cell lines, severe hypoxemia, CD4 lymphocyte count of 349 cells/mm^3^, and no other organic compromise. Tomographic findings are shown in Figure 1. 

Based on clinical and radiological pattern, the initial diagnostic impression was pneumonia by common microorganisms, SARS-CoV-2 or *Pneumocystis jirovecii*; and treatment was started with Trimethoprim Sulfamethoxazole, Dexamethasone, and Cefepime. The patient presented a torpid evolution, respiratory deterioration progressing to acute respiratory distress syndrome, and required invasive mechanical ventilation. 

Initial microbiologic studies are shown in Table 1. All of them were negative except for a positive latex cryptococcal antigen agglutination test in peripheral blood. It has been documented that this antigenic test could present cross-reactivity false-positives in other infections by microorganisms, such as *non-neoformans Cryptococcus*, *Trichosporon* spp., and *Capnocytophaga canimorsus*; and in non-infectious conditions, such as rheumatoid factor positivity [15,16]. Nonetheless, the previous meningeal infection was well-documented with cerebrospinal fluid culture confirmation, basal ganglia hyperintense lesions, and high cerebrospinal fluid opening pressure; so the probability of a true-positive is highly likely. The HIV status of the patient was also confirmed negative in three separate occasions, using two 4-generation tests (ELISA) and a viral load.

**Figure 2:**
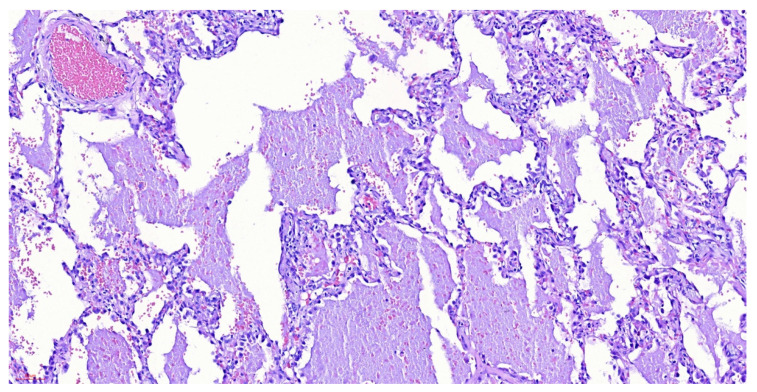
Wedge lung biopsy with hematoxylin-eosin stain, demonstrating pulmonary parenchyma with emphysematous and reactive epithelial changes, pneumocyte hyperplasia, and dilated alveoli occupied by eosinophilic material, with a low-grade hemorrhage and occasional histiocytes and anthracosis. Negative for malignancy, changes related to alveolar proteinosis.

Fiberoptic bronchoscopy was performed, with an only positive finding of abundant hyaline secretion without endobronchial lesions. Bronchoalveolar lavage revealed a positive molecular test for *M. tuberculosis* (MBT) and abundant mesothelial cells of reactive appearance, histiocytes, and lymphocytes. Biopsy of the right lower lobe was performed which revealed changes suggestive of alveolar proteinosis; however, due to an insufficient sample, the patient underwent wedge lung biopsy confirming the diagnosis (Figure 2). 

After ruling out viral disease, common bacteria, and other opportunistic microorganisms, antibiotic treatment was suspended and Isoniazid, Rifampicin, Ethambutol, and Pyrazinamide (HRZE) were started. Regarding the alveolar proteinosis, he was taken to whole lung lavage twice, with a progressive resolution of respiratory symptoms. Hematologic and autoimmune causes were ruled out due to reports of normal bone marrow studies, absence of connective tissue disease manifestations and a negative immune profile, respectively. Unfortunately, we did not measure anti-granulocyte and macrophage colony-stimulating factor antibodies (anti-GM-CSF) to diagnose idiopathic pulmonary proteinosis, so considering the adequate response to management with HRZE, successful extubation, and resolution of respiratory distress in two weeks, we considered MBT as the trigger for alveolar proteinosis. The patient reported a progressive decrease on shortness of breath, and a significant improvement on quality of life. He continues using supplementary oxygen to date.

## 3. Discussion

Some opportunistic infections cause transient CD4+ lymphocytopenia, and their treatment has been related to immune recovery. This transient phenomenon has been described in infections associated with an immune-response mainly mediated by T lymphocytes and mononuclear phagocytic cells, especially with the CD4+ subset of lymphocytes [17,18]. However, this immune response also requires a cytokine release network activation. In acute infection, levels of Interleukin-1β (IL-1B), soluble interleukin-2 receptor (sIL-2R), interleukin-6 (IL-6), and tumor necrosis factor-α (TNF-a) increase [1]. It is hypothesized that this activation conversely reduces the CD4+ count, and increases the CD8+ count, reducing the CD4+/CD8+ ratio. Proper treatment directly modulates the cytokine network activation, restoring the CD4+/CD8+ population ratio and normalizing the CD4+ count. The role of cytokine response as a regulator of T-cell differentiation under these circumstances is not yet fully understood, but these events may have clinical implications as transient immunosuppressive periods [3].

Kony et al. [17] report that up to 14% of patients with tuberculosis, HIV-negative, and no other immunosuppressive conditions have CD4 counts below 300 cells/mm^3^. Luo et al. [18], demonstrated that treatment directed at this population was associated with an increase in CD4+ lymphocyte count, as well as a decrease in levels of IL-1B, sIL-2R, IL-6 and TNF-a. Similarly, cryptococcal infection has been consistently correlated with CD4+ lymphocytopenia; however, to date, treatment of cryptococcosis has not been reported to improve lymphocyte counts. 

Another relevant aspect is the finding of alveolar proteinosis, which cannot be confirmed as idiopathic or secondary due to absence of anti-GM-CSF measurement tests. In most cases, infection by MBT is favored by the structural and immunological changes observed in alveolar proteinosis (mainly macrophage dysfunction) [19]. However, it has also been proposed that alveolar proteinosis is the consequence of infections by microorganisms, including MBT infection, which pathophysiologically can be explained by the stimulation of type II pneumocytes by the mycobacteria, increasing the secretion of pulmonary surfactant and favoring the development of proteinosis [19]. Similarly, it has been described that a decrease in GM-CSF correlates with a higher risk of developing tuberculosis. Alveolar proteinosis could be associated with other infectious conditions, such as cryptococcal post-infectious inflammatory response syndrome (PIIRS) [20], or infections due to *Nocardia* spp., *Pneumocystis* spp., *Acinetobacter* spp., *Aspergillus* spp., and *Cladosporium* spp. [8]. However, PIIRS usually presents at the induction or consolidation phases of cryptococcal management and requires corticosteroid treatment for improvement [20]. Other microbiological studies were negative with no evidence of microorganisms other than MBT, and, after antituberculous treatment, the patient presented rapid clinical improvement with progressive resolution of alveolar proteinosis. Other non-infectious conditions that could present with a similar radiological pattern include sarcoidosis, non-specific interstitial pneumonia and diffuse alveolar hemorrhage. Drug reaction with eosinophilia and systemic symptoms (DRESS) syndrome has been described to cause interstitial infiltrates along with other features such as bilateral nodules or pleural effusion [21]. All of these non-infectious differential diagnoses were ruled out after laboratory results and histopathologic studies. 

The present case would be among the few cases reported since 1979 to date [13], in which alveolar proteinosis is described because of MBT infection. Literature describes two patterns: The first in which MBT infection is documented before the diagnosis of alveolar proteinosis with a negative report of anti-GM-CSF; and the second in which the measurement of anti-GM-CSF is not available, but antituberculous treatment leads to a decrease in proteinosis and favorable clinical outcomes [3,4,5,6,7,9,10,11,13,14]. Our case belongs to the second pattern of presentation; however, to our knowledge it would be the first case reporting alveolar proteinosis secondary to *M. tuberculosis* in a patient with transient CD4 lymphocytopenia and cryptococcosis. 

## 4. Conclusions

This case is relevant to the literature, demonstrating how cryptococcosis treatment is related to an increase in lymphocyte count. Likewise, this immune reconstitution and a concomitant infection by *M. tuberculosis* could aid the development of alveolar proteinosis, with a favorable resolution after antituberculous treatment. 

## Figures and Tables

**Table 1:** Microbiologic studies.

Laboratory	Specimen	Result
Galactomannan	Blood	Negative
*Histoplasma* urinary antigen	Urine	Negative
Respiratory FilmArray BioFire^®^ (Adenovirus, Coronavirus 229E-HKU1-NL63-OC43, Human Metapneumovirus, Human Rhinovirus/Enterovirus, Influenza A-B, Parainfluenza virus 1-2-3-4, Respiratory Syncytial Virus, *Bordetella parapertussis* (IS1001), *Bordetella pertussis* (ptxP), *Chlamydia pneumoniae*, *Mycoplasma pneumoniae*)	Sputum	Negative
Microbiological culture (Tryptic Soy Broth media with and without CO_2_)	Sputum and blood	Negative
Gram stain	Sputum and blood	Negative
Bacilloscopy (serial)	Sputum	Negative
*Mycobacterium tuberculosis* PCR (1081 and 6110 genes with TaqMan probe)	Sputum	Negative
*Mycobacterium tuberculosis* culture (Löwenstein-Jensen media)	Sputum	Negative
RT-PCR for SARS-CoV-2 (RdRp, N and E genes)	Nasopharyngeal swab	Negative
Cytomegalovirus viral load	Blood	Negative
HIV antibodies 1/2 (4 generation ELISA)	Blood	Negative
HIV Viral load	Blood	Negative
Latex Cryptococcal antigen	Blood	Positive
Antinuclear antibodies	Blood	Negative
Autoantibodies to extractable nuclear antigens	Blood	Negative
Complement (C3, C4)	Blood	Normal
Rheumatoid factor	Blood	Negative
Anti-Scl-70 Antibodies	Blood	Negative

## Data Availability

All relevant information has been presented in the case report. Any additional data may be made available on reasonable request from the corresponding author.

## Abbreviations

Anti-GM-CSF	Anti Granulocyte Macrophage Colony-Stimulating Factor antibodies
DRESS	Drug reaction with eosinophilia and systemic symptoms
ELISA	Enzyme-linked Immunosorbent Assay
GM-CSF	Granulocyte Macrophage Colony-Stimulating Factor
HRZE	Isoniazid, Rifampicin, Ethambutol, Pyrazinamide
IL-1β	Interleukin-1β
MBT	*Mycobacterium tuberculosis*
PCR	Polymerase Chain Reaction
PIIRS	Cryptococcal post infectious inflammatory response syndrome
RT PCR	Reverse Transcription - Polymerase Chain Reaction
SARS-CoV2	Severe Acute Respiratory Syndrome Coronavirus 2
